# Differences in the Impact of Heart Rate Variability on the Surgical Approach in Patients With Early Cervical Cancer: Laparoscopic versus Open Surgery

**DOI:** 10.3389/fonc.2022.804242

**Published:** 2022-06-03

**Authors:** Jian Liu, Jingfeng Wang, Zhaoya Deng, Shiqi Liu, Guangqiao Li, Yilin Sun, Longfei Gao, Chenghui Li, Bo Shi

**Affiliations:** ^1^Department of Gynecologic Oncology, First Affiliated Hospital, Bengbu Medical College, Bengbu, China; ^2^School of Medical Imaging, Bengbu Medical College, Bengbu, China; ^3^Anhui Key Laboratory of Computational Medicine and Intelligent Health, Bengbu Medical College, Bengbu, China

**Keywords:** early cervical cancer, autonomic nerve, heart rate variability, laparoscopic surgery, open surgery

## Abstract

**Background:**

Evidence suggests that the risk of recurrence and death in patients with early cervical cancer (ECC) undergoing minimally invasive surgery is significantly higher than that in patients undergoing open surgery. However, the mechanisms underlying such a difference remain unclear. Heart rate variability (HRV) represents autonomic nerve activity, which is related to tumorgenesis and can be used as a prognostic indicator for various cancers. The main purpose of this study was to explore the difference in the effects of laparoscopic and open surgery on HRV in ECC patients.

**Methods:**

A total of 68 ECC (FIGO IA1 with lymphovascular space invasion -IIA2) patients undergoing radical hysterectomy for the first time (84% open group vs. 16% laparoscopic group) were included. A single-lead micro-ECG recorder was used to collect 5 min electrocardiograms 1 day before the operation and 3 days after the operation, and then HRV time domain and frequency domain indices were analyzed, including mean heart rate (MeanHR), maximum heart rate (MaxHR), minimum heart rate (MinHR), the standard deviation of all normal-to-normal intervals (SDNN), the root mean square of successive interval differences (RMSSD), very low-frequency power (VLF), low-frequency power (LF), high-frequency power (HF), total power (TP), and the ratio of LF to HF (LF/HF).

**Results:**

Heart rate (i.e., MeanHR, MaxHR, and MinHR) were significantly higher, and HRV (i.e., SDNN, RMSSD, LF, HF, and TP) were significantly lower after the operation than before the operation in both the laparoscopic and open groups (*P* < 0.05). The postoperative reduction in RMSSD and HF was significantly higher in the laparoscopic group than in the open group (*P* < 0.05).

**Conclusions:**

These data suggest that radical hysterectomy can lead to increased heart rate and decreased HRV in patients with ECC, which can negatively affect cardiac autonomic regulation. Compared with open surgery, laparoscopic surgery has a greater negative impact on the HRV of ECC patients.

## Introduction

Cervical cancer (CC) is one of the most common malignant tumors in gynecology, and its morbidity and mortality rank fourth in females worldwide (1). According to the guidelines of the National Comprehensive Cancer Network and the European Society of Gynecological Oncology, radical hysterectomy (laparoscopic surgery or open surgery) is the standard treatment for early cervical cancer (ECC) patients (International Federation of Gynecology and Obstetrics (FIGO) IA-IIA) (2). After surgical treatment, the primary tumor of most ECC patients is removed, which can minimize the tumor load, achieve a better curative effect, and prolong the survival time (3, 4).

Some retrospective studies have shown that no significant differences in the recurrence rate and survival rate have been observed between minimally invasive surgery and open surgery in patients with ECC and locally advanced stage (5–8). In addition, compared with open surgery, laparoscopic surgery has certain advantages, such as less intraoperative blood loss, a lower wound infection rate, a shorter hospital stay and fewer postoperative complications (9–11). Nevertheless, the findings reported in these studies have been questioned due to small sample sizes, short follow-up durations, and a lack of adjustment for potential confounding factors (12). A recent multicenter prospective randomized controlled trial on laparoscopic treatment of ECC demonstrated that compared with open surgery, laparoscopic surgery for ECC patients has a lower disease-free survival rate and overall survival rate and a higher local recurrence rate and mortality rate (13). Additionally, Melamed et al. used the US national database (the National Cancer Database and the Surveillance, Epidemiology, and End Results (SEER) 18-registry database) to compare the survival differences of CC patients undergoing different surgeries. The results showed that during the median follow-up period of 45 months, the 4-year mortality was 9.1% among patients who underwent laparoscopic surgery and 5.3% among those who underwent open surgery (14). Since then, experts have agreed that laparoscopic surgery should no longer be advocated (15). However, the relevant mechanisms leading to this difference remain unknown.

The systemic stress response caused by surgical trauma can affect the autonomic nervous system (ANS) (16). Autonomic disorders not only increase the incidence of postoperative cardiovascular complications but also promote the growth and spread of tumor cells (17–19). Other studies also observed that certain associations between ANS function and disease recurrence and prognosis. In a large epidemiological study, patients with gastric ulcers who underwent vagotomy showed an increased incidence of colonic polyps during long-term follow-up (20). Erin et al. found that inhibition of vagus nerve can increase tumor metastasis in mice with breast cancer (BC) (21). Barron et al. indicated tha BC patients taking β-blockers (propranolol) had a lower specific mortality than patients not taking β-blockers (hazard ratio, 0.19; 95% CI, 0.06 to 0.60) (22). Heart rate variability (HRV), a noninvasive biomarker reflecting the ANS, has been widely used for the evaluation of prognosis in patients with a variety of cancers (23, 24). For example, De Couck et al. showed that compared with healthy people, cancer patients were often accompanied by damage to autonomic nerves, resulting in decreased HRV (25). Arab et al. found that the more advanced the tumor stage of BC patients, the more significant the decrease of HRV time domain parameters (26). Hu et al. demonstrated that the decrease of HRV in patients with gastric cancer predicts an increase in the severity of the tumor. The authors confirmed that HRV was associated with clinical stage, tumor size, tumor invasion, lymph node metastasis and distant organ involvement (27). Furthermore, a recent systematic review of HRV and cancer prognosis indicated that low HRV was associated with shorter survival, higher tumor burden, and worse prognosis (28). As a result, using HRV to detect the autonomic changes that may occur in ECC patients undergoing laparoscopic and open surgery may be able to better explain the differences in survival observed between the two methods. The main purpose of this paper is to test two hypotheses by comparing the effects of two surgical procedures (open surgery/laparoscopic surgery) on HRV in patients with ECC. Hypothesis 1: The HRV of ECC patients decreases significantly after the operation; that is, the operation has a negative effect on the prognosis of ECC patients. Hypothesis 2: Compared with open surgery, laparoscopic surgery has a greater negative impact on the HRV of ECC patients.

## Methods

### Subjects

This study was prospective clinical controlled trials. The study participants included 72 patients with CC treated in the Department of Gynecology and Oncology, the First Affiliated Hospital of Bengbu Medical College, from November 2020 to May 2021. These patients underwent radical hysterectomy as a primary therapy. Surgical approach (laparoscopic surgery or open surgery) was determined on an individual basis with each patient after discussion of the risks and benefits of both options. All procedures were performed in accordance with the ethical standards outlined in the 1964 Declaration of Helsinki and its later amendments. All patients were informed of the experimental details and signed informed consent forms. The study was approved by the Medical Ethics Committee of the First Affiliated Hospital of Bengbu Medical College (Bengbu, Anhui, China) (registration number: 2021KY010).

### Inclusion and Exclusion Criteria

The inclusion criteria were as follows: (1) ECC diagnosed by pathological examination; (2) pathological types: squamous cell carcinoma, adenocarcinoma and adenosquamous carcinoma; (3) new patients without surgery, radiotherapy or chemotherapy; (4) met the indications for CC radical surgery and treated with radical hysterectomy; (5) tumor stages IA1 with lymphovascular space invasion -IIA2, based on the guidelines of FIGO 2018; and (6) operative methods: laparoscopic radical hysterectomy + pelvic lymph node dissection (laparoscopic group) or open radical hysterectomy + pelvic lymph node dissection (open group). The exclusion criteria were as follows: (1) pregnancy; (2) patients with primary diseases of the heart, liver, kidney; (3) patients with other tumors; and (4) patients with unqualified clinical stages.

### Data Collection

A single-lead electrocardiogram (ECG) recorder (version 2.8.0, Healink-R211B, Healink Ltd., Bengbu, China) was used to collect 5 min supine ECG data from ECC patients at 1 day before the operation and 3 days after the operation. The sampling rate of the ECG recorder was set to 400 Hz, and the signal bandwidth was set to 0.6–40 Hz. All patients were requested to breathe as smoothly as possible in a quiet environment during the measurement. The V6 lead was used, and the measuring electrodes were Ag/AgCl disposable electrodes (JunKang Ltd., Shanghai, China). The environment was kept quiet, and the room temperature was maintained at 23 ± 1°C.

### Heart Rate Variability Analysis

The QRS complex in the ECG signal was detected using the Pan-Tompkins algorithm, from which an R-R interval time (RRI) series was obtained (29). The time-varying threshold algorithm was used to automatically correct the artifacts caused by extraction technology, interference and ectopic heartbeat (30). Then, the HRV data were analyzed by time domain and frequency domain methods The main parameters included mean heart rate (MeanHR), maximum heart rate (MaxHR), minimum heart rate (MinHR), standard deviation of all normal-to-normal intervals (SDNN), root mean square of successive interval differences (RMSSD), very low-frequency (VLF: 0–0.04 Hz) power, low-frequency (LF: 0.04–0.15 Hz) power, high-frequency (HF: 0.15–0.40 Hz) power, total power (TP: 0–0.4 Hz) power and the ratio of LF power to HF power (LF/HF). Prior to frequency analysis, the RRI time series was resampled at 4 Hz using cubic spline interpolation (31).

Among these parameters, SDNN and TP reflect overall variability. HF is related to RMSSD, and both are adopted as markers of vagal modulation, whereas LF is considered representative of the modulation of both sympathetic and parasympathetic nerves (23, 32, 33). LF/HF is an indicator of the balance between sympathetic and vagal nerves (23, 32).

HRV analysis was performed using Kubios HRV Premium software (version 3.1.0, https://www.Kubios.Com, Kubios Oy, Kuopio, Finland) (34).

### Statistical Analysis

Data were first tested for normality using the Shapiro-Wilk normality test. If the data showed a normal distribution, paired *t*-tests were used to examine the difference in HRV parameters before and after the operation; otherwise, nonparametric Wilcoxon signed-rank tests were used. Cohen’s *d* was used to evaluate the effect sizes. The differences in HRV parameters before and after the operation between the two groups were compared by independent sample *t*-tests. All statistical analyses were performed using SPSS, version 23.0 (IBM Corp., Chicago, Illinois, United States of America). *P* <  0.05 was taken as statistically significant.

## Results

A total of 72 ECC patients were enrolled in this study. We further excluded several cases due to poor ECG quality (*N* = 1), ectopic heartbeats > 10% of all beats (*N* = 1) or due to abnormal data extremes (*N* = 2). Therefore, data from 68 cases were analyzed in the current study. Open radical hysterectomy was performed in 57 cases (84%) and laparoscopic radical hysterectomy in 11 cases (16%). **Table 1** shows the basic data of the studied ECC patients. There were no significant differences in patient characteristics such as age, body mass index (BMI), diabetes, menopausal status, FIGO stage, lymph node metastasis, pathological type, tumor size and invasion depth between the laparoscopic and open groups (all *P* > 0.05).

**Table 1:** Basic data of the CC patients studied.

	Open group (*N* = 57)	Laparoscopic group (*N* = 11)	*p* value
Age (years)	52.4 ± 9.3	46.8 ± 11.6	0.082
BMI (kg/m^2^)	24.4 ± 3.0	24.7 ± 3.8	0.741
Diabetes, N (%) yes no	1 (1.8) 56 (98.2)	1 (9.1) 10 (90.9)	0.299^c^
Menopausal status, N (%) yes no	36 (63.2) 21 (36.8)	5 (45.5) 6 (54.5)	0.446^b^
FIGO stage, N (%) IA1-IB3 IIA1-IIA2	42 (73.7) 15 (26.3)	9 (81.8) 2 (18.2)	0.849^b^
Lymph node metastasis, N (%) yes no	8 (14.0) 49 (86.0)	1 (9.1) 10 (90.9)	1.000^b^
Histological type, N (%) SCC AC	48 (84.2) 9 (15.8)	9 (81.8) 2 (18.2)	1.000^b^
Tumor size, N (%) <4 cm ≥4 cm	38 (66.7) 19 (33.3)	9 (81.8) 2 (18.2)	0.523^b^
Depth of invasion, N (%) <1/2 mm ≥1/2 mm	27 (47.4) 30 (52.6)	8 (72.7) 3 (27.3)	0.123^a^

a Stands for the Pearson’s chi squared test.

b Stands for the chi square test for continuity correction.

c Stands for the Fisher exact test.

N, number of individuals; BMI, body mass index; SSC, squamous cell carcinoma; AC, adenomatous carcinoma. FIGO, International Federation of Gynecology and Obstetrics.

**Figure 1:**
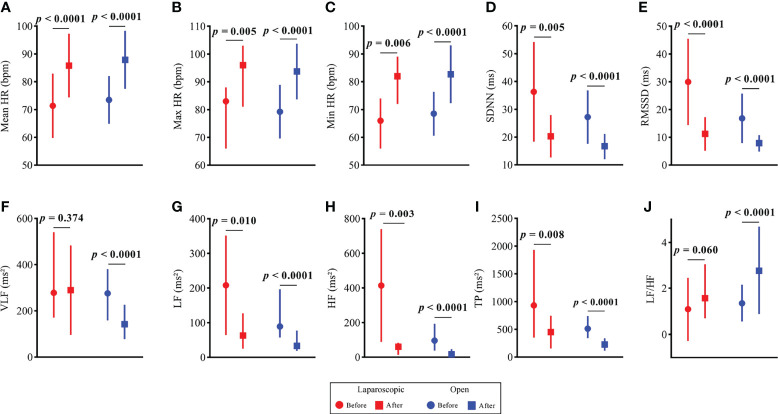
HR and HRV results of the open group and laparoscopic group before vs. after radical hysterectomy. **(A)** Mean HR; **(B)** Max HR; **(C)** Min HR; **(D)** SDNN; **(E)** RMSSD; **(F)** VLF; **(G)** LF; **(H)** HF; **(I)** TP and **(J)** LF/HF.

**Figure 2:**
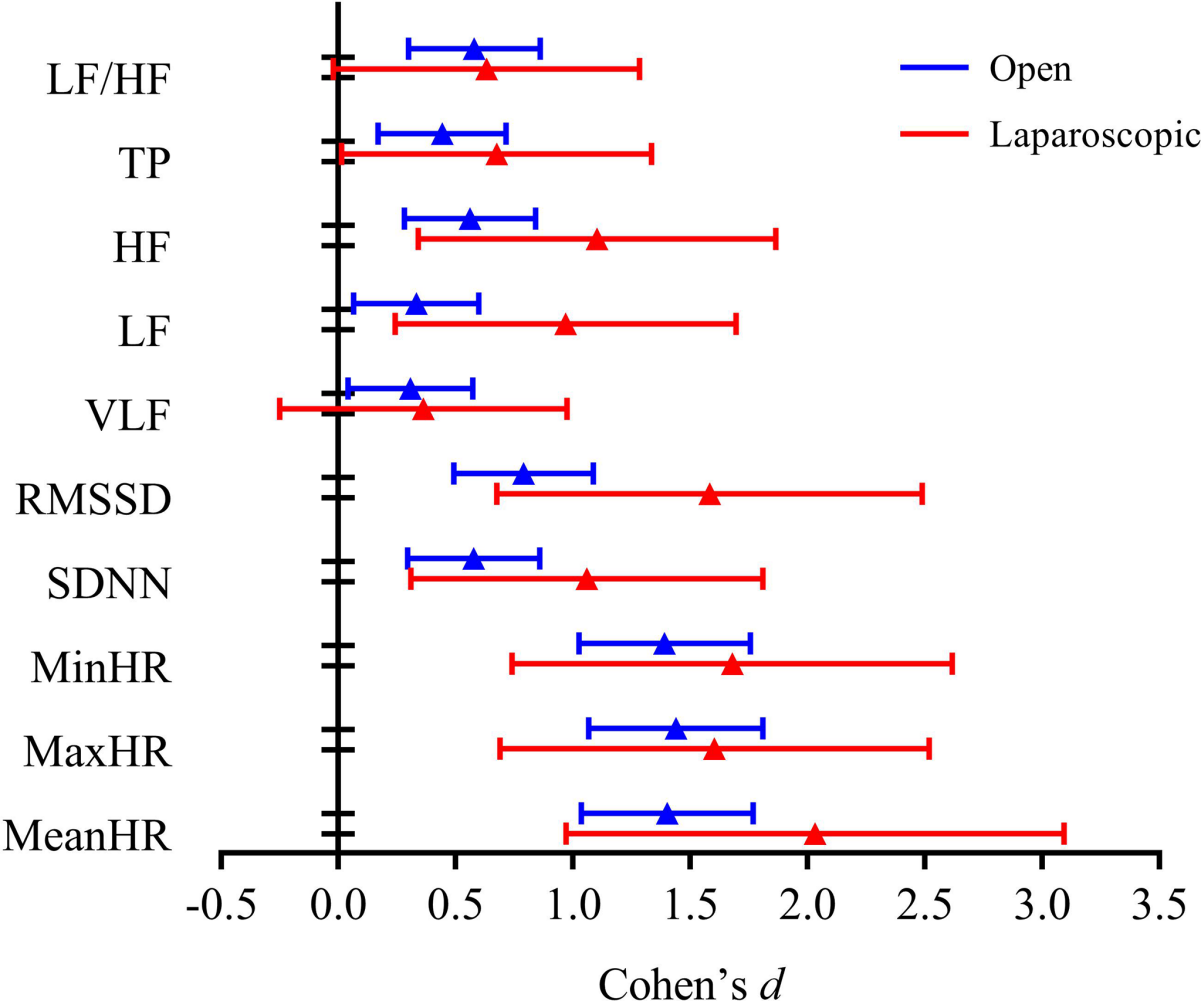
Effect size of all HR and HRV indices in the open group and laparoscopic group before vs. after radical hysterectomy.

**Figure 3:**
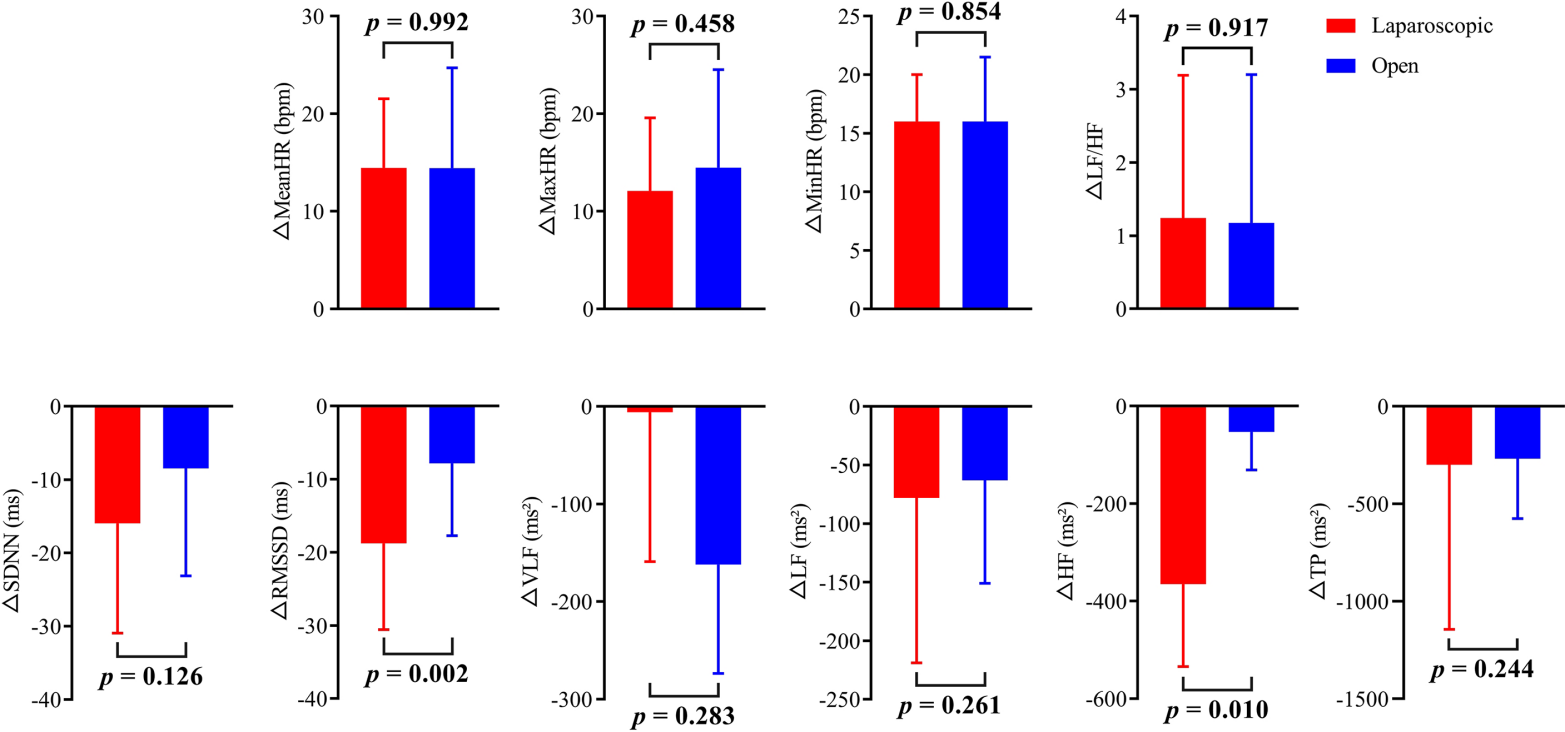
The change in HR and HRV is indicated by the value after radical hysterectomy minus the value before radical hysterectomy for both the open group and laparoscopic group.

The HR and HRV results of the laparoscopic group and open group after radical hysterectomy compared with before are summarized in **Figure 1**. The postoperative HR (i.e., MeanHR, MaxHR, and MinHR) was significantly higher than the preoperative HR in both the laparoscopic and open groups (*P* < 0.05). The postoperative HRV (i.e., SDNN, RMSSD, LF, HF, and TP) was significantly lower than the preoperative HRV in the two groups (*P* < 0.05). A significant increase in LF/HF and a significant decrease in VLF were observed in the open group, while LF/HF and VLF showed no statistically significant difference in the laparoscopic group. The effect sizes of all HR and HRV indices in the laparoscopic group and open group before and after radical hysterectomy are shown in **Figure 2**. In addition, the differences in HR and HRV, indicated by the postoperative value minus the preoperative value, were compared in the laparoscopic group and open group (**Figure 3**). The reduction in RMSSD (laparoscopic group = -18.7 versus open group= -7.8) and HF (laparoscopic group = -365 versus open group = -53) after the operation was significantly higher in the laparoscopic group than in the open group (*P* = 0.002, *P* = 0.010 respectively).

## Discussion

A preliminary study of 68 patients with ECC who underwent radical hysterectomy revealed that HR was significantly higher and the HRV parameters (SDNN, RMSSD, LF, HF, and TP) were significantly lower after the operation than before the operation in both the laparoscopic and open groups. Furthermore, the difference in RMSSD and HF before and after the operation in the laparoscopy group was significantly higher than that in the open group. Thus, this study directly suggested that radical hysterectomy can lead to increased HR and decreased HRV in patients with ECC. Compared with open surgery, laparoscopic surgery has a greater negative impact on the HRV of ECC patients.

Radical hysterectomy is the mainstay of treatment for ECC, but surgery will inevitably cause certain psychological and physical damage to patients. Previous studies showed that HRV could be used to quantify surgical stress response and predict postoperative complications and prognosis. For example, Gogenur et al. confirmed that the HRV and the ANS in surgical patients would develop circadian disturbance, resulting in an increase in short-term and long-term morbidity and mortality of cardiovascular disease (16). Ushiyama et al. found that HRV decreased significantly on postoperative day 1 and day 7 after digestive surgery and HRV indices correlated significantly to operation time, blood loss and operative complications (35). Several studies have also investigated the impact of surgical treatment on HRV in cancer patients. For instance, Amar et al. showed that HRV significantly decreased in cancer patients within the first week after major abdominal or chest surgery (36). Hansen et al. found that BC patients still had alterations in autonomic tone and decreased HRV and experienced the loss of circadian rhythm 14 days after tumor resection (37). Our research was consistent with these results. Surgery results in autonomic nerve injury and a significant decrease in HRV parameters.

The complex interaction between the ANS, endocrine system and immune system is enhanced by surgical trauma. First, surgical trauma as a source of stress will stimulate the sympathetic nerve and hypothalamus-pituitary-adrenal cortex axis, trigger the body’s stress response, and lead to sympathetic excitement and cortisol release (38). It was found that the level of cortisol was positively correlated with the severity of the stress response (39). Cortisol can also further enhance the sympathetic-mediated cardiovascular response to stress, ultimately resulting in an increase in HR (40). Second, surgical trauma can cause a series of inflammatory reactions, which affect immune function (41, 42). Some studies have shown that the levels of C-reactive protein (CRP) and interleukin-6 (IL-6) increase within 14 days after abdominal surgery (43). The vagus nerve can inhibit inflammation through the cholinergic anti-inflammatory pathway and regulate the function of immune cells (44, 45). Consequently, the decrease of vagus nerve activity after surgery may be related to the increase of inflammatory factors and the decrease of immune function. The present study found that regardless of open or laparoscopic surgery, the HR of ECC patients 3 days after the operation was higher than that at 1 day before the operation, and most of the HRV indices 3 days after the operation were lower than those at 1 day before the operation, which was basically consistent with the above mechanism. This result suggests that both open and laparoscopic surgery can cause damage to autonomic function in patients with ECC. Further studies are needed to clarify the correlation between postoperative HRV and inflammatory factors and immune function indicators.

However, it is worth noting that the impact of open and laparoscopic surgery on the HRV of ECC patients was slightly different. The postoperative LF/HF of the patients undergoing open surgery was significantly higher and the postoperative VLF was significantly lower after the operation than before the operation, while the postoperative LF/HF and VLF of patients undergoing laparoscopic surgery were not significantly different. A recent report argued that postoperative pain stimulation could cause a significant increase in LF/HF (46). Compared with open surgery, laparoscopic surgery uses artificial ports for intra-abdominal operation, which reduces the trauma and pain stimulation of traditional laparotomy on abdominal wall structure of patients. At the same time, compared with traditional surgery, laparoscopic surgery causes minimal blood loss and leads to lower wound infection rate, decreasing the level of sympathetic stimulation and the effects on LF/HF. Currently, the physiological mechanism of VLF is poorly defined, but it has been proven in recent years that VLF has the characteristics of slow recovery after abnormal stimulation (47). No statistically significant difference was detected between the preoperative and postoperative VLF of patients undergoing laparoscopic surgery, which might be because patients who underwent laparoscopic surgery recovered from surgical stimulation faster than patients who underwent open surgery.

Moreover, we found that the reduction in RMSSD and HF after the operation was significantly higher in the laparoscopic group than in the open group, which means that laparoscopic surgery has a greater negative impact on the HRV of ECC patients. One possible reason is the effect of CO2 pneumoperitoneum. During laparoscopic surgery, CO2-induced positive pressure pneumoperitoneum has adverse effects on cardiac hemodynamics, including the decrease of venous return and cardiac output, and the increase of systemic vascular resistance, pulmonary artery pressure and central venous pressure (48–50). These hemodynamic effects can cause a reflex increase in baroreceptor activity, combined with the direct mechanical effect of CO2 intraperitoneal inflation through diaphragmatic and phrenic nerve stretching and the negative cardiac effect of hypercapnia, which together lead to the increase of sympathetic nerve activity and the decrease of vagus nerve activity (51–54). This may have a relevant impact on patient HRV. RMSSD and HF indicate the vagal nerve tone. The vagal nerve has been proposed to be involved in the regulation of tumor progression. The active vagus nerve has an inhibitory effect on inflammation, oxidative stress and excessive sympathetic activity (55–57). Cancer patients often experience vagus damage, which manifests as a decrease in HRV, and the lower the HRV is, the worse the prognosis (58). Giese-Davis et al. demonstrated that in patients with recurrent or metastatic BC, a higher HF was closely related to longer overall survival (59). Bijoor et al. found that compared with healthy controls, the RMSSD of early and advanced cancer patients decreased significantly. Compared with patients with early cancer (TNM I and II), those with advanced cancer (TNM III and IV) had significantly lower RMSSD (60). These results showed that the lower vagus activity indicated by HRV significantly predicted a worse prognosis. Therefore, the difference in prognosis between laparoscopic and open surgery may be attributed to the difference in vagus nerve activity. This research seems to provide a theoretical basis for the conclusion that the risk of recurrence and death after laparoscopic surgery in ECC patients is significantly higher than that after open surgery. However, The mechanism of laparoscopic and open surgery leading to differences in vagus nerve activity still needs further investigation.

The limitation of this study was that it did not consider the impact of HR changes on HRV. This was because separating the influence of HR on HRV from the influence of surgical methods on HRV would require a large number of subjects, and the number of laparoscopic surgery cases in our study was relatively small. In addition, several possible confounders such as operation time, intraoperative blood loss and intraoperative and postoperative complications were not adjusted, which might affect the difference in HR and HRV before and after operation. Furthermore, more background variables, such as physical activity, stress levels, use of medications, and other relevant medical variables, could not be included. Thus, further studies with larger sample sizes, more detailed background variables and adjustment for relevant confounders should be performed to validate the results of the present study.

In summary, radical hysterectomy, whether performed *via* open or laparoscopic surgery, will cause the decrease of HRV and the damage of autonomic function in patients with ECC. Compared with open surgery, laparoscopic surgery has a greater negative impact on the HRV of ECC patients.

## Data Availability Statement

The raw data supporting the conclusions of this article will be made available by the authors, without undue reservation.

## Ethics Statement

The studies involving human participants were reviewed and approved by the Medical Ethics Committee of the First Affiliated Hospital of Bengbu Medical College (Bengbu, Anhui, China). The patients/participants provided their written informed consent to participate in this study.

## Author Contributions

Conceptualization, BS and JL; methodology, JW and GL; formal analysis, GL and JW; resources, BS and JL; data curation, JL, YS, and LG; writing—original draft preparation, BS, SL, and CL; writing—review and editing, BS, JW, and ZD; supervision, JL; project administration, BS; funding acquisition, BS. All authors have read and agreed to the published version of the manuscript.

## Funding

This research was funded by the “512” Outstanding Talents Fostering Project of Bengbu Medical College (grant number BY51201312) and the Science Research Project of Bengbu Medical College (grant number 2021byzd057).

## Conflict of Interest

A direct family member of BS owns stock in HeaLink Ltd., Bengbu, China.

The remaining authors declare that the research was conducted in the absence of any commercial or financial relationships that could be construed as a potential conflict of interest.

## Publisher’s Note

All claims expressed in this article are solely those of the authors and do not necessarily represent those of their affiliated organizations, or those of the publisher, the editors and the reviewers. Any product that may be evaluated in this article, or claim that may be made by its manufacturer, is not guaranteed or endorsed by the publisher.
